# Effects of Blending Tobacco Lignin with HDPE on Thermal and Mechanical Properties

**DOI:** 10.3390/ma15134437

**Published:** 2022-06-23

**Authors:** Venkata Gireesh K. Menta, Irfan Tahir, Abdulaziz Abutunis

**Affiliations:** Department of Mechanical & Industrial Engineering, University of Minnesota Duluth, Duluth, MN 55812, USA; tahir037@d.umn.edu (I.T.); abutu001@d.umn.edu (A.A.)

**Keywords:** lignin, polyethylene, tobacco lignin, blends, characterization, biopolymers

## Abstract

Depletion of fossil fuels and the detrimental environmental impacts of synthetic plastics have prompted a global interest in bio-based polymers. Lignin is an abundant, unused, and low-value byproduct of pulping and biochemical operations that has the potential to decrease the need for plastics derived from petroleum. Melt blending is one of the easiest strategies for expanding the commercial applications of lignin. Concerns remain, however, regarding the negative effects of lignin on the final composite material’s performance, and the increase in manufacturing costs. This study investigates the effects of blending lignin extracted from tobacco using a novel one-step processing technique on injection molding parameters, and the mechanical, physical, and thermal properties of high-density polyethylene (HDPE). By extruding HDPE pellets and lignin powder, varying blend concentrations (0, 5, 10, 15, and 30% wt.) were produced. Scanning electron microscopy (SEM) and optical microscopy were used to investigate the compatibility of the blend morphology. Results indicated that interfacial interactions were achieved as particles of tobacco lignin were well dispersed and uniformly distributed throughout HDPE. Intermolecular interactions between HDPE and lignin were also discovered through Fourier-transform infrared (FTIR) spectral analyses. The tensile test results showed that increase in lignin content up to 15% wt. had little effect on tensile strength, but at 30% wt., a 19% reduction was observed. With the addition of 5, 10, 15, and 30% wt. of lignin, the tensile modulus increased by 4%, 29%, 25%, and 8%, respectively. TGA results demonstrated that at 15% and 30% wt., tobacco lignin acted as a thermal stabilizer. The processability study revealed that tobacco lignin could be processed easily using injection molding without requiring significant changes to the process parameters. Overall, tobacco lignin showed great promise as a biodegradable HDPE filler.

## 1. Introduction

The production of petroleum-based plastics is extremely energy- and emission-intensive. In 2015, the production of plastic in the United States alone contributed more than 10 million tons of carbon dioxide equivalents (CO_2_e) to the atmosphere. About 108 million metric tons of CO_2_e are emitted annually by plastic production outside the United States, where petroleum is the primary fuel source [1]. Plastic waste or incineration causes additional greenhouse gas emissions and environmental harm. In 2019, over 348 million tons of plastics were produced globally, of which 50% were for single-use purposes and 8 million tons were discarded into the oceans [2]. As plastic production is projected to increase at a compound annual growth rate (CAGR) of 3.7% between 2020 and 2025, plastic pollution in marine and terrestrial environments is a major concern [3]. Consequently, it is imperative to find ways to reduce plastic production and consumption. Fuels and feedstocks based on renewable and carbon-neutral energy plant sources are attractive alternatives. However, using cultivated crops intended for animal or human consumption adds upward pressure on food availability, prices, and arable land. Lignin, a biopolymer that is abundantly available, underutilized, and a low-value by-product of pulping and biochemical processes, is an excellent non-food source alternative. Derived from the Latin word lignum, which means wood, lignin is an organic polymer that is needed for the formation of plant cell walls and is found in the plant’s support tissues [4]. It is the second most abundant renewable carbon source on Earth, after cellulose [5]. Approximately 50 million tons of lignin are produced annually as a byproduct of paper pulping industries, of which, only 2% is commercially utilized [6].

One of the easiest and inexpensive ways of using lignin is by melt blending with other plastics. Several researchers have studied the effects of blending lignin with plastics [7,8,9,10,11]. Alexy et al. studied the impact of lignin addition to low-density polyethylene (LDPE) on processing stability, mechanical characteristics, and thermal properties [12]. The authors discovered that the mechanical characteristics of blends deteriorated consistently with increasing lignin concentration and that lignin worked as a thermal stabilizer at higher concentrations. A decrease in flexural strength and modulus was observed by Peng et al. when polypropylene (PP) was blended with lignin, wood flour, and cellulose [13]. Similar trends of deterioration of mechanical properties with the addition of lignin have led to studies by multiple researchers exploring alternative ways to improve the performance of lignin blends [14,15,16,17,18,19,20,21].

Sailaja et al. and Lv et al. reported that the addition of a compatibilizer significantly improved the thermal stability and mechanical properties, almost approaching values close to neat LDPE [15,16]. Esterification and free-radical grafting enhanced the mechanical and thermal characteristics of HDPE/lignin blends, according to Hu et al. and Faurk et al. [17,18]. Almost all the studies show improvement in the properties of blended composites when secondary modifications, such as esterification, alkalization, and co-grafting were used [19,20,21]. However, scalability and increased processing costs hinder using this approach on an industrial scale. To increase the prospects for lignocellulosic biomass in value-added products, it is crucial to find a solution that does not compromise either performance or cost. Producing lignin without expensive secondary processes helps keep costs down.

The inconsistency of lignin’s characteristics, even when employing the same source material and method, is another well-known challenge. One of the major causes of the variability and poor performance of lignin can be found in the recovery methods employed. Currently, commercially available lignin is obtained from processes where cellulose and hemicellulose are the main products while lignin is a byproduct. Hence, little attention is paid to the quality of the lignin. Processes dedicated to obtaining quality lignin have the potential to address several of the reported issues. In the present work, lignin produced via such processes without any secondary modifications is utilized.

At least three US states have traditionally relied heavily on the tobacco industry for employment (North Carolina, Georgia, and Kentucky). This research aims to reduce the production and consumption of environmentally hazardous petroleum-based plastics by utilizing byproducts from the production of harmful tobacco. In addition, lignin extracted from tobacco plants is of particular interest given that no research has been published on its effects when combined with plastics. The physical, mechanical, and thermal effects of blending lignin extracted from tobacco stalks with high-density polyethylene (HDPE) are reported and discussed.

## 2. Materials and Methods

### 2.1. Materials

HDPE pellets from M. Holland, Inc., (Northbrook, IL, USA) were utilized. The density of HDPE pellets was 0.84 g/cm^3^, and the melt flow index (MFI) was 0.70 g/10 min. Lignin extracted from tobacco in the form of powder using a proprietary one-step butanol-based organosolv process was obtained from Attis Innovations, Milton, GA, USA. No additives were added to the lignin powder. Both materials were vacuum dried at 60 °C for 9 h to eliminate any moisture. Materials were stored in a vacuum-sealed container.

### 2.2. Preparation of Blends

**Figure 1:**
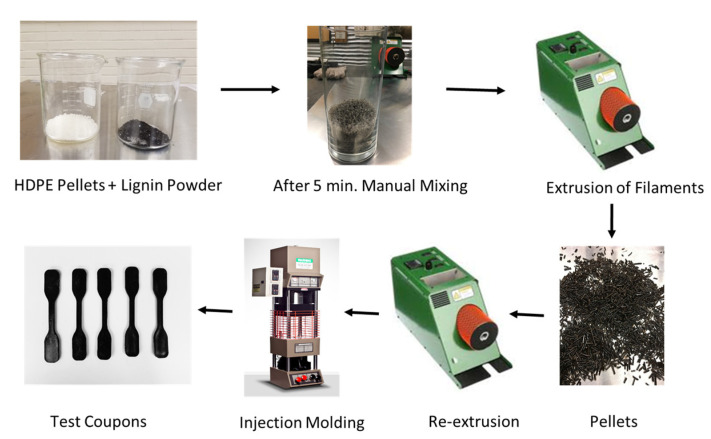
Schematic illustration of sample preparation.

Different proportions of HDPE pellets and tobacco lignin powder (5, 10, 15, 30% wt.) were weighed separately and placed in a container for 5 min of rigorous manual mixing. The mixtures were then compounded in a Filabot EX2 single screw extruder (Barre, VT, USA) at approximately 0.9 kg per hour and 175 °C. The utilized extrusion parameters are listed in Table 1 below. Extrudates were pelletized and re-extruded for a second time to achieve better blending. The filaments were then re-pelletized and kept in vacuum-sealed containers for injection molding. The process of specimen preparation is depicted in Figure 1 below as a schematic diagram.

### 2.3. Injection Molding Test Coupons

The ASTM D638 Type IV standard dog-bone tensile test coupons were produced utilizing a Morgan Press G-125T injection molding machine from Morgan Industries, Inc., Long Beach, CA, USA [22]. Initially, the process parameters that produced defect-free, short-shot, and flash-free HDPE parts were determined via a trial and error process. The same parameters were then used for lignin blends and modified as needed. Table 2 shows the processing parameters used for neat HDPE and HDPE/lignin blends. While most of the processing parameters remained constant, the injection pressure needed to be increased with the increase in lignin content. Without the additional pressure and resultant pressure gradient at the mold gate, the molten plastic was not able to fill the molds. At 30% wt. lignin loading, it was also necessary to increase the barrel and nozzle temperature. With the exception of these minor processing modifications, HDPE plastic coupons were manufactured without difficulty at all, even with 30% wt. tobacco lignin loading and without additives. As a result, the HDPE/tobacco lignin blend materials showed considerable promise in commercial extrusion and injection molding applications without the need for extensive and costly fabrication process modifications.

### 2.4. Characterization

#### 2.4.1. Fourier-Transform Infrared Spectroscopy (FTIR)

The FTIR spectra of the blend samples were acquired on a Thermo Fisher Scientific Nicolet iS50 FTIR Spectrometer (Waltham, MA, USA) using diamond ATR in the scan range from 4000 to 400 cm^−1^ with a resolution of 4 cm^−1^ following the ASTM E1421 standard [23]. Each specimen was subjected to three repetitions.

#### 2.4.2. Differential Scanning Calorimetry (DSC)

Using a TA Instruments Discovery DSC 250 from Thermo Fisher Scientific, Waltham, MA, USA, and in compliance with ASTM D3418-15, thermal characterization of the blends was conducted [24]. Approximately 5 mg of samples were heated from 25 °C to 180 °C at a rate of 10 °C/min, held at this temperature for 5 min to erase thermal history, cooled to 25 °C at a rate of 10 °C/min, and again heated to 180 °C at the same rate (the second heating scan). Each specimen was subjected to three repetitions. 

#### 2.4.3. Thermogravimetric Analysis (TGA/DTG)

TA Instruments Discovery TGA 550 Thermo Fisher Scientific, Waltham, MA, USA, was utilized to conduct thermogravimetric analysis in accordance with ASTM E1131 [25]. Under a nitrogen environment at a heating rate of 10 °C/min from 25 °C to 600 °C, samples of 15–20 mg of HDPE and HDPE/lignin blends were examined. Once the mass loss plateau was reached, the environment in the furnace was changed from inert oxygen to reactive oxygen at 600 °C and then heated to 800 °C at a rate of 10 °C/min. Three repetitions were carried out for each sample.

#### 2.4.4. Scanning Electron Microscopy (SEM) and Optical Microscopy

Using a Joel JSM-6590LV Scanning Electron Microscope (SEM) from JEOL, Ltd., Akishima, Tokyo, Japan, equipped with an INCA X-act EDS detector for elemental analysis, cross-sections of the test specimens were studied. The surfaces of the samples were observed in a low vacuum environment (30 Pa) using an accelerating voltage of 20.0 kV. The samples were bonded to aluminum stubs. A Leica DVM6 optical microscope from Leica Microsystems, Inc., Deerfield, IL, USA., was also utilized to analyze lignin particle size distribution at 100–2000X magnification to acquire a better understanding of the blend morphology and its relationship to the mechanical properties of the blend samples. 

#### 2.4.5. Physical Testing

Density and specific gravity of the samples were measured as per ASTM D792 using a Mettler Toledo precision balance to investigate the effects of lignin addition on the physical properties of the blends [26]. At least, five specimens were tested for each blend. 

#### 2.4.6. Mechanical Testing

The tensile properties of the blends were determined at room temperature using MTS Universal testing equipment from MTS Systems, Eden Prairie, MN, USA, in accordance with ASTM D638 and a crosshead speed of 5 mm/min. The values for the ultimate tensile strength (UTS), Young’s modulus, and the strain at rupture were measured. According to ASTM D785-08, hardness tests were conducted using a PTC Instruments (Los Angeles, CA, USA) shore D hardness tester [27]. At least five specimens were tested for each blend, and the mean results with standard deviation were reported.

## 3. Results

### 3.1. Fourier-Transform Infrared Spectroscopy Analysis

**Figure 2:**
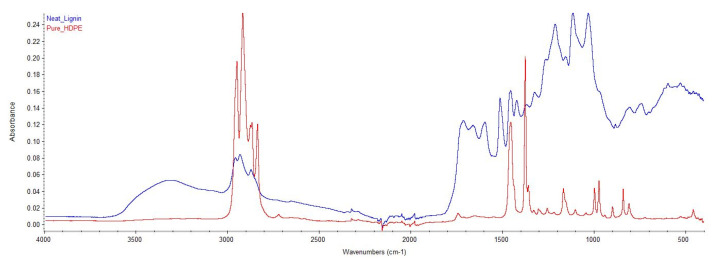
FTIR spectra of HDPE and lignin.

The FTIR spectra of neat tobacco lignin and HDPE used in this study are shown in Figure 2. The chemical structure of lignin used in the blend highly influences the resulting properties of the blends. Tobacco lignin showed bands at 1065 and 1120 cm^−1^, indicating the presence of the syringyl (S) ring. The band at 1717 cm^−1^ corresponded to the carbonyl groups, while the presence of conjugated carbonyl groups is evident by the band at 1667 cm^−1^. All lignin samples presented a broadband in the 3000–3600 cm^−1^ region corresponding to OH stretching vibrations. The band observed at 2938 cm^−1^ can be predominantly due to CH stretching in the methyl and methylene groups, and at 2850 cm^−1^ because of stretching in the methoxyl groups of side chains in lignin. Moreover, a band was observed at 1428 cm^−1^ because of aromatic skeletal vibration combined with C-H in-plane deformation. Strong bands were also observed at 1600 and 1513 cm^−1^, which were due to aromatic skeletal vibration and can be seen as evidence of the splitting of aliphatic side chains in lignin and/or condensation reactions. From the Neat HDPE FTIR spectrum, a symmetric C-H stretching was seen by the band at 2980 cm^−1^, an asymmetric CH_2_ stretching at 2920 cm^−1^, a symmetric CH_2_ stretching at 2825 cm^−1^, CH_2_ scissoring at 1462 cm^−1^, CH_2_ wagging at 1367 cm^−1^, and a symmetric CCH bending at 1160 cm^−1^.

**Figure 3:**
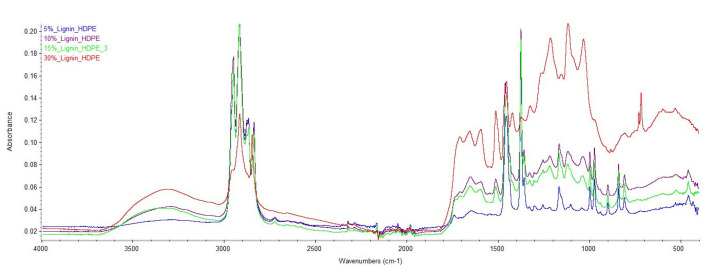
FTIR spectra of HDPE/lignin blends.

Figure 3 shows the FTIR spectra of blends with different lignin content. The absorption bands in the 3000–3600 cm^−1^ region are due to −OH stretching and are visible in all the blends. With an increase in the lignin content, an increase in the intensity of −OH stretching was observed. These hydroxyl groups are typically phenolic hydroxyl groups with a strong ability to form hydrogen bonds with carbonyl groups. Significant changes in this region can be understood as phenolic hydroxyl groups of lignin interacting with carbonyl groups of HDPE. The shift of the bands to lower wavenumbers further asserts this observation and can be taken as evidence of the formation of intermolecular hydrogen bonds [28]. 

### 3.2. Differential Scanning Calorimetry

**Figure 4:**
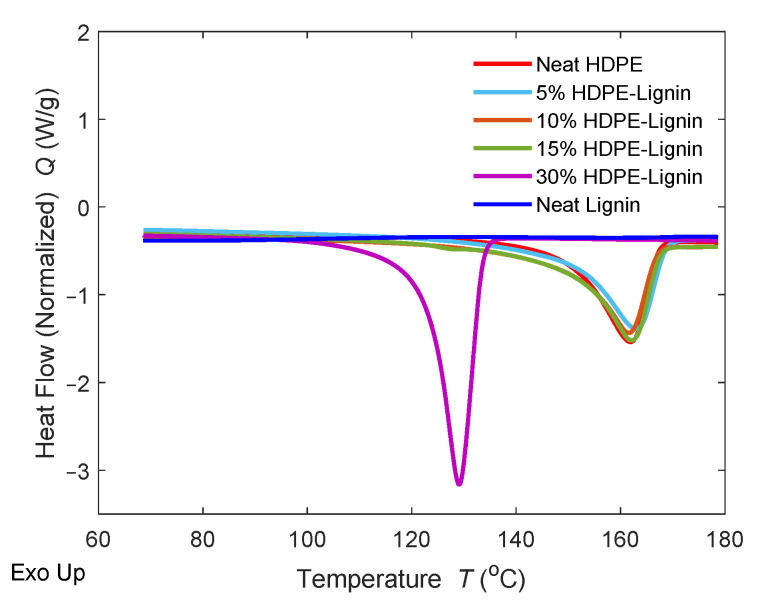
The DSC traces of HDPE/lignin blends recorded during the first heating scan.

The DSC thermograms of HDPE, pure lignin, and blends are presented in Figure 4. DSC is an acceptable method for determining the glass transition temperature (T_g_) of lignin; however, lignin’s complex chemical structure makes it challenging to detect its T_g_ [29]. The T_g_ of lignin typically ranges from 90 to 180 °C. The T_g_ of lignin could not be determined in this instance. Table 3 lists the crystallization temperature (T_c_), the melting temperature (T_m_), the exothermic enthalpy of crystallization (H_c_), and the endothermic enthalpy of melting (H_m_) of the blends. As shown in Table 3 and Figure 4, the melting temperature of HDPE deviated slightly on blending with 5% wt. of lignin but remained almost unchanged on increasing the amount of lignin to 10 and 15% wt. However, an 18.75% drop is observed at 30% wt. lignin loading. Similar behavior was observed with T_c_, as evident in Figure 4. Moreover, both the exothermic and endothermic enthalpies significantly increased when the lignin percentage was increased from 15 to 30% wt. The unique behavior at 30% wt. lignin loading is consistent in both thermal and mechanical tests. 

### 3.3. Thermogravimetric Analysis

**Figure 5:**
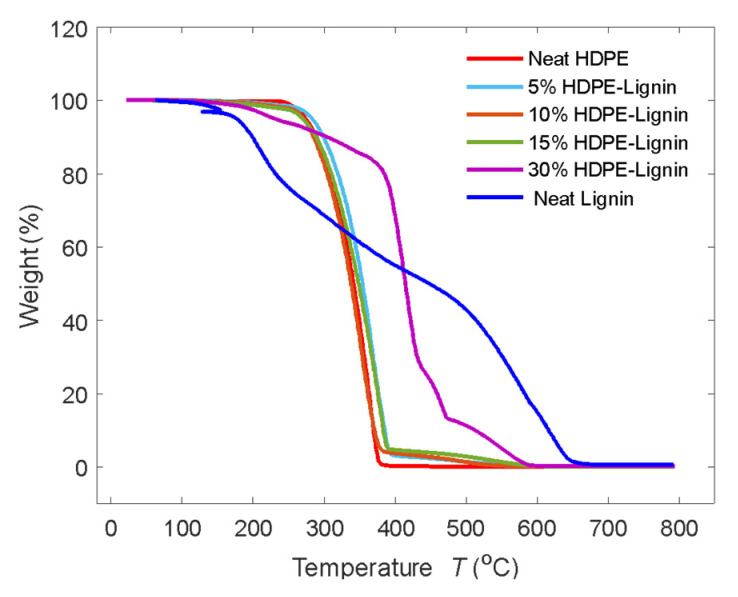
TGA curves for different blends of HDPE and lignin.

**Figure 6:**
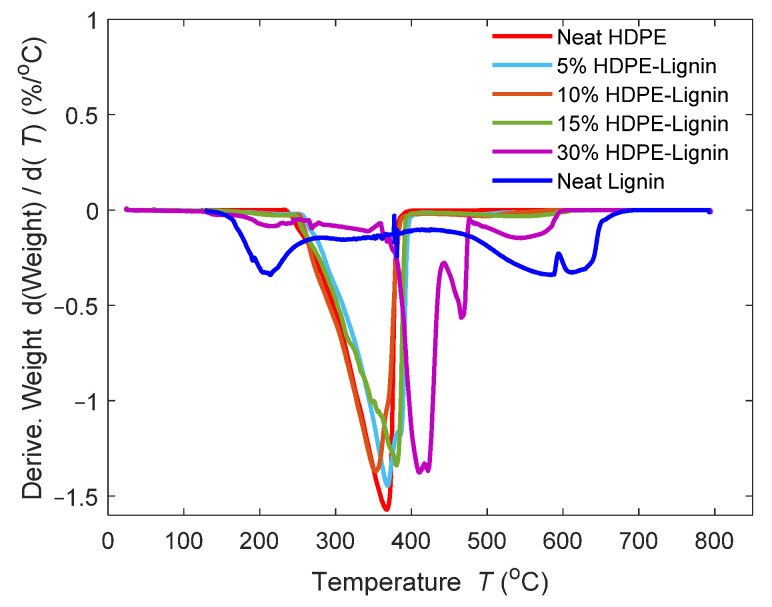
DTGA curves of different blends of HDPE and lignin.

Thermogravimetric analysis (TGA) was performed to study the effects of lignin on the thermal stability of the blends. Figure 5 and Figure 6 show the TGA and the DTGA curves for all the samples. A typical one-stage decomposition was observed for all the samples except for the 30% wt. HDPE–lignin blend. Not only does 30% wt. have a much higher onset temperature, but also a much higher decomposition peak temperature 30% wt. blend achieves maximum decomposition at 437.53 °C, which is 56.57 °C more than that of neat HDPE, and it starts decomposing at 380.29 °C, which is 69.04 °C higher than that of neat HDPE. This sudden increase in thermal stability of the blend material as lignin content is increased can be explained due to the charring nature of lignin, which acts as an insulating layer around HDPE. Similar observations were made by Marchovich et al. with lignin demonstrating the same effect up to 450 °C [30]. While using lignin as a filler does provide an advantage for the thermal stability of the blends, it also increases the residual mass, which fails to burn completely. This can be seen especially in neat lignin, where 30.49% of the material was left to burn and did not completely burn until 550 °C. Following this, it can be said that at lower concentrations, lignin has a destabilizing effect on the thermal degradation of blends, but at higher concentrations (30% wt.), tobacco lignin acts as a thermal stabilizer. Table 4 shows the onset temperature, end temperature, decomposition peak temperature, and residual mass at 550 °C. Figure 6 shows the DTGA curves from which the decomposition peak temperature can be calculated. As shown in Table 4, the decomposition peak temperature remained close to the neat HDPE. 

### 3.4. Scanning Electron Microscopy (SEM) and Optical Microscopy

**Figure 7:**
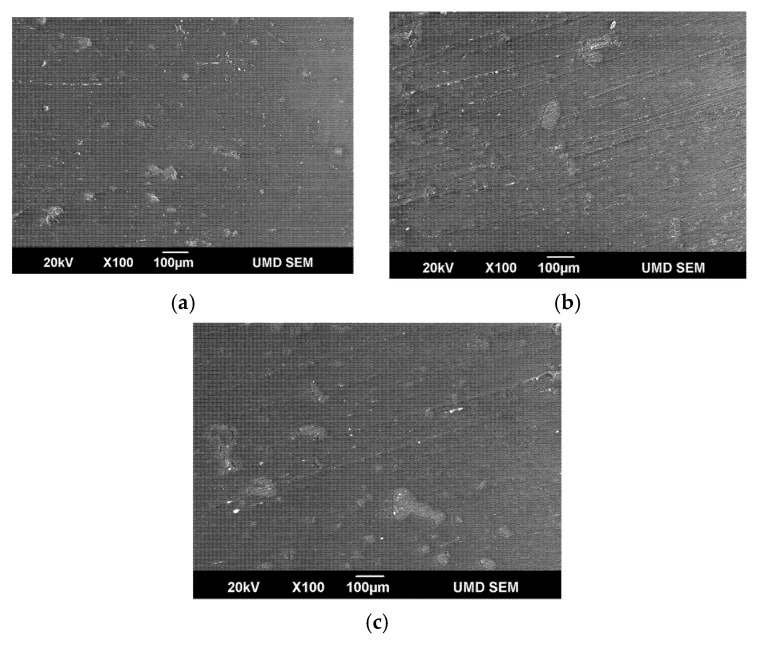
SEM micrograph of (**a**) 10% wt., (**b**) 15% wt., and (**c**) 30% wt. lignin and HDPE blends.

Figure 7 displays the SEM micrographs of 10% wt., 15% wt., and 30% wt. blends. At 5% wt., SEM scans revealed hardly any particles, indicating excellent dispersion. When lignin content was increased to 10% wt., lignin particles seemed to have diffused uniformly into HDPE. At 30% wt., however, larger aggregates were found. Typically, the strong polarity of lignin leads to agglomeration, especially at high-loading concentrations, and the mechanical characteristics decrease as the degree of agglomeration increases. Smaller lignin particles imply that lignin was successfully dispersed in HDPE even though a single screw extruder was used. 

Optical microscopy was employed to examine the lignin particle distribution in HDPE since dispersion plays a significant effect on the mechanical performance of blends. Lignin particle sizes were quantified by measuring the grain diameter, and the data were then analyzed to form a probability density function for the grain sizes, which is given by Equation (1).

(1) $$f\left(x,\mu ,\;\sigma \right)=\frac{1}{\sigma \sqrt{2\pi }}\;e^{\frac{-{\left(x-\mu \right)}^{2}}{2\sigma^{2}}}$$

where *x* is the continuous random variable, *μ* is the mean, *σ* is the standard deviation, and *σ*^2^ is the variance. 

**Figure 8:**
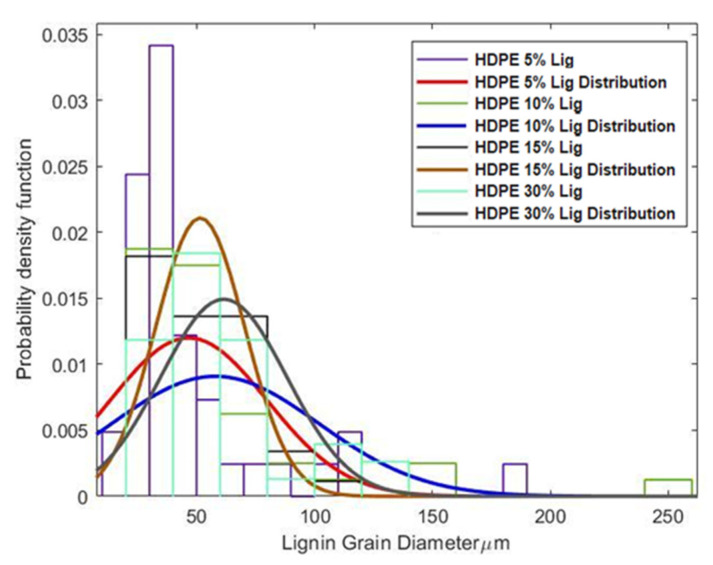
Frequency distribution showing lignin particle size.

A histogram created from the sample data (Figure 8) represents overall lignin grain sizes in diameter (μm) and their frequencies in the specimen. For all categories of blends, the particle sizes ranged from 10 μm all the way up to 150 μm. The normal distribution shows that, in all blend samples, the average peak was around 50–75 μm. Such small particle sizes were only reported for secondarily modified lignin. For example, 55 μm of grain diameter was reported for acetylated lignin [13]. However, the butyrated lignin has better particle sizing at 14 μm and a better distribution than tobacco lignin, but butyration adds a high cost to the process. From the above comparison, it can be easily perceived that tobacco lignin has excellent compatibility without the need for expensive modifications. 

### 3.5. Density

**Figure 9:**
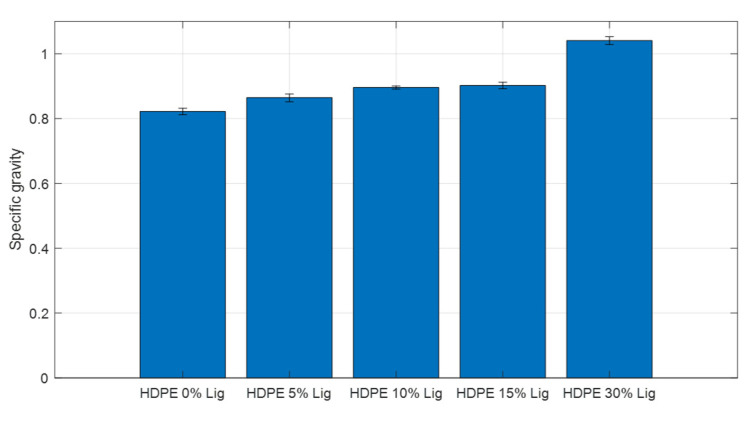
Specific gravity comparison for HDPE–lignin blend compositions.

The specific gravity values of the HDPE–lignin blends are displayed and compared in Figure 9. Density and specific gravity consistently increased as lignin was added. Neat HDPE showed a density of 840 kg/m^3^. Density increased by 2.3%, 5.9%, 7.1%, and 23%, with the increase of lignin by 5, 10, 15, and 30% wt., respectively. The gradual increase in the density can explain the need for increasing the pressure during the injection molding process. That also agrees with the increase in crystallization observed during the DSC tests. 

### 3.6. Tensile Tests

**Figure 10:**
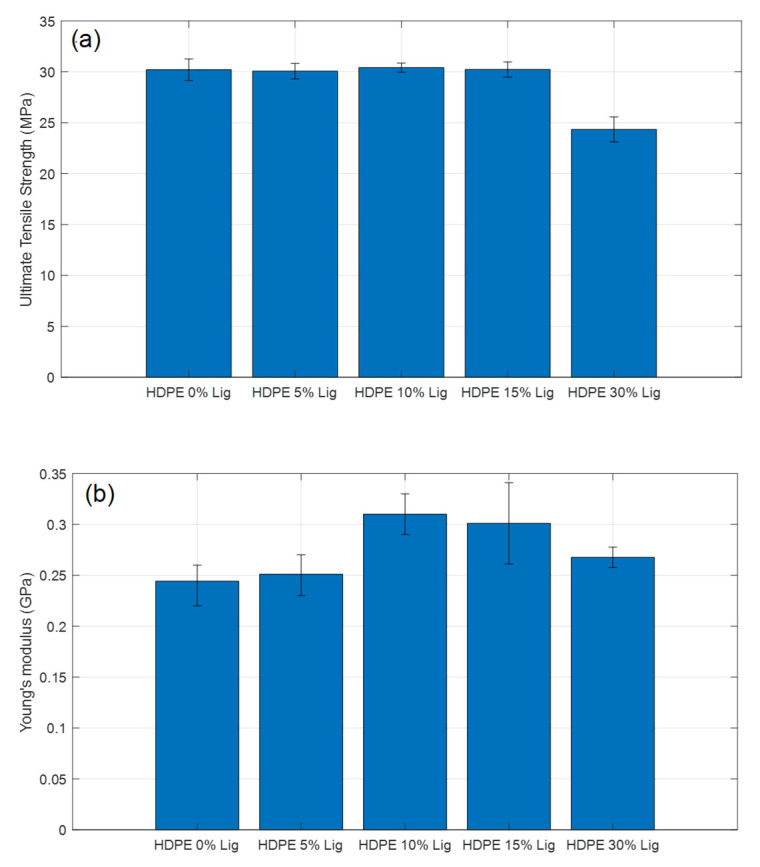
(**a**) Ultimate tensile strength and (**b**) Young’s modulus comparison of HDPE–lignin blend materials.

Figure 10 compares the tensile strength and modulus values of the HDPE lignin blends. With an addition of 5% wt. of lignin, the tensile strength dropped slightly by 2.6%. However, the decrease is within the margin of error. Similar results were observed for 10 % wt. and 15% wt. as shown in Figure 10a. Hence, it can be concluded that statistically, the tensile strength remained unaffected by the addition of lignin up to 15% wt. However, higher lignin loading (30% wt.) resulted in a 19% reduction in tensile strength. Tensile modulus increased by 4%, 29%, 25%, and 8% with the addition of 5, 10, 15, and 30% wt. of tobacco lignin to the HDPE (Figure 10b). Young’s modulus improvement can be attributed to a robust interface between lignin particles and HDPE, allowing better load transfer. 

The sheer volume of polyphenols, quinone structures, and hydroxyl groups in the lignin molecule makes it easier to aggregate, resulting in poor mechanical properties. Good compatibility results in smaller lignin particle sizes and improved mechanical performance. As shown in Figure 8, a particle size of around 50–60 μm was observed at 5%, 10%, and 15% wt. lignin. At 30% wt., larger agglomerates were observed. Table 5 compares the tensile strength, stiffness, and strain values for different blends. At 5%, 10%, and 15% wt. lignin loadings, the tensile strength almost remained constant while a 19% reduction was observed at 30% wt. lignin loading. These results are in agreement with the findings from SEM and DSC. 

Most studies found that adding just 10% wt. of lignin reduced tensile strength by as much as 48% and as little as 3.7%, as shown in Table 6. Secondary treatments, such as esterification and acetylation, are commonly employed to increase compatibility and, thus, mechanical performance. Sailaja et al. [15] and Dehne et al. [20] reported a reduction of 16.66% and 13.04% in tensile strength with esterification and acetylation processes, respectively. In the current study, however, a slight increase of tensile strength by 0.69% at 10% wt. lignin, and reduction by 19.40% at 30% wt. lignin was observed. Without any secondary modifications, the tobacco lignin obtained in this work presented superior performance compared to the modified lignins obtained from other sources in the literature. The significant improvement in the mechanical performance can be attributed to the uniform and enhanced dispersion of lignin particles as seen in the SEM images.

### 3.7. Shore D Hardness 

**Figure 11:**
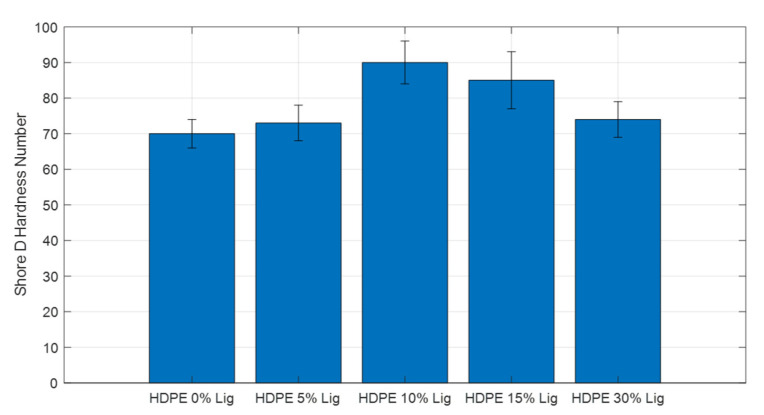
Shore D hardness test comparison for all blend materials.

Figure 11 displays the Shore D hardness numbers for different specimens of blended materials. In every instance, lignin added to composites increased their hardness. The highest values were obtained for the biocomposites with 10% wt. lignin. Results are in close agreement with the findings from SEM, DSC, and tensile testing. 

## 4. Conclusions

The processability and physical and mechanical properties of HDPE/unmodified tobacco lignin (5%, 10%, 15%, and 30% by weight) prepared by melt mixing were investigated. The addition of tobacco lignin did not affect the injection molding processing parameters, implying that it does not add any cost to the processing. DSC and FTIR indicated the formation of intermolecular hydrogen bonds. SEM and optical microscopy further showed that lignin particles were well dispersed and homogeneously distributed in HDPE using only a single screw extruder. Compared to the values reported in the literature, the unmodified tobacco lignin particles were around 55 μm, similar to the values reported for esterified and acetylated lignins. It is well known that dispersion and particle size are directly related to the mechanical properties of the blends. Accordingly, no reduction in tensile strength values was observed except at 30% wt. Tensile stiffness and strain to failure increased in all cases of lignin addition. The UTS values were compared to those reported in the literature, and it was observed that the unmodified tobacco lignin shows performance comparable to esterified and acetylated lignins. On the basis of physical, mechanical, and thermal properties, a 15% lignin addition produced optimal behavior. The study further ascertained that developing extraction processes with the quality of lignin as the main focus can result in a promising biodegradable material that can reduce petroleum-based plastic consumption. 

## Figures and Tables

**Table 1:** Extrusion process parameters.

Extrusion Parameters	Specifications
Feed screw and drive:	35 RPM
Nozzle diameter:	2.85 mm
Pellet size:	3.18 mm
Drive force:	9.6 Nm
Compression ratio:	2:5:1
L/D ratio:	12

**Table 2:** Injection molding parameters for varying lignin content.

Blend Composition	Barrel Temp (°F)	Nozzle Temp (°F)	Clamping Pressure (psi)	Plate Temp (°F)	Injection Pressure (psi)	Injection/ Dwell Time (s)
Neat HDPE	3000	390	400	8.6	7000	2
5 % wt.Lignin	3500	390	400	8.6	7000	2
10 % wt. Lignin	4000	390	400	8.6	7000	2
15 % wt. Lignin	4500	390	400	8.6	7000	2
30 % wt. Lignin	5000	395	420	8.6	7000	2

**Table 3:** DSC test results.

Blend Composition	T_c_ (°C)	Exothermic Enthalpy (J/g)	T_m_ (°C)	Endothermic Enthalpy (J/g)
Neat HDPE	117.16 ± 1.32	108.04 ± 5.66	160.59 ± 0.89	107.38 ± 7.77
5% wt. Lignin	120.09 ± 4.22	87.49 ± 6.98	161.88 ± 0.40	82.65 ± 8.49
10% wt. Lignin	119.05 ± 3.22	87.15 ± 4.38	160.81 ± 0.11	86.91 ± 2.88
15% wt. Lignin	118.45 ± 2.54	95.40 ± 6.99	160.58 ± 0.63	95.96 ± 9.25
30% wt. Lignin	116.03 ± 0.07	140.80 ± 11.5	129.43 ± 0.03	142.37 ± 13.23

**Table 4:** TGA test results.

Blend Composition	T_onset_ (°C)	T_End_ (°C)	Decomposition Peak Temperature (°C)	Residual Mass at 600 °C (%)
Neat HDPE	311.25	376.80	366.27	0.018
5% wt. Lignin	309.54	389.41	367.36	0.060
10% wt. Lignin	302.29	375.30	352.79	0.065
15% wt. Lignin	313.31	386.84	379.85	1.334
30% wt. Lignin	380.29	437.53	422.84	4.996
Neat Lignin	103.58	647.67	418.93	16.221

**Table 5:** Comparison of tensile properties of HDPE–lignin blend.

Blend Composition	Ultimate Tensile Strength (MPa)	Young’s Modulus (GPa)	Strain at Break (mm/mm)
Neat HDPE	30.20 ± 1.05	0.24 ± 0.02	0.61 ± 0.13
5% wt. Lignin	29.42 ± 0.76	0.25 ± 0.02	0.47 ± 0.11
10% wt. Lignin	30.41 ± 0.44	0.31 ± 0.02	0.37 ± 0.03
15% wt. Lignin	30.22 ± 0.74	0.301± 0.03	0.32 ± 0.03
30% wt. Lignin	24.34 ± 1.23	0.2676 ± 0.02	0.23 ± 0.04

**Table 6:** Comparison of UTS for HDPE–lignin blends in related works.

	Materials	Change in UTS % with the Increase of Lignin Content in Blend	
		10% wt. PE/Lignin	30% PE/Lignin
**Present work**	HDPE-tobacco lignin	0.69% increase	19.40% decrease
**Earlier works**			
P. Alexy et al. [12]	LDPE—lignin	3.70% decrease	55.55% decrease
R. Sailaja et al. [15]	LDPE—esterified lignin with phthalic anhydride	-	16.66 % decrease
Laura Dehne et al. [20]	Acetylated lignin	17.3% decrease	39.13% decrease
	Propionated lignin	10.86% decrease	13.04% decrease
	Butyrated lignin	4.34% decrease	13.04% decrease
R. Pucciariello et al. [31]	LLDPE	73.47% decrease	-
	LDPE	75% decrease	
	HDPE	47.82% decrease	
J. Sameni et al. [32]	Non-wood soda lignin	2.85% increase	8.1% increase
	5% MAPE added	5.40% increase	12.5% increase
F. Luo et al. [33]	Non compatibilized lignin	4.76% decrease	-
Kharade et al. [14]	HDPE–lignin	37.76% decrease	52.63% decrease

## Data Availability

Not applicable.
